# Study on Deterioration Process of Magnesium Oxychloride Cement under the Environment of Dry–Wet Cycles

**DOI:** 10.3390/ma16051817

**Published:** 2023-02-22

**Authors:** Chenggong Chang, Lingyun An, Jinmei Dong, Weixin Zheng, Jing Wen, Fengyun Yan, Xueying Xiao

**Affiliations:** 1 State Key Laboratory of Advanced Processing and Recycling of Non-Ferrous Metals, Lanzhou University of Technology, Lanzhou 730050, China; 2 Key Laboratory of Comprehensive and Highly Efficient Utilization of Salt Lake Resources, Qinghai Institute of Salt Lake, Chinese Academy of Sciences, Xining 810008, China; 3 Key Laboratory of Salt Lake Resources Chemistry of Qinghai Province, Xining 810008, China; 4 Qinghai Provincial Key Laboratory of Nanomaterials and Technology, College of Physics and Electronic Information Engineering, Qinghai Minzu University, Xining 810007, China

**Keywords:** magnesium oxychloride cement, dry–wet cycles, microstructure, phase composition, mechanical properties, deterioration process

## Abstract

To reveal the deterioration process of magnesium oxychloride cement (MOC) in an outdoor, alternating dry–wet service environment, the evolution of the macro- and micro-structures of the surface layer and inner core of MOC samples as well as their mechanical properties and increasing dry–wet cycle numbers were investigated by using a scanning electron microscope (SEM), an X-ray diffractometer (XRD), a simultaneous thermal analyser (TG-DSC), a Fourier transform infrared spectrometer (FT-IR), and an microelectromechanical electrohydraulic servo pressure testing machine. The results show that as the number of dry–wet cycles increases, the water molecules gradually invade the interior of the samples, causing the hydrolysis of P 5 (5Mg(OH)_2_·MgCl_2_·8H_2_O) and hydration reactions of unreacted active MgO. After three dry–wet cycles, there are obvious cracks on the surface of the MOC samples, and they suffer from warped deformation. The microscopic morphology of the MOC samples changes from a gel state and a short, rod-like shape to a flake shape, which is a relatively loose structure. Meanwhile, the main phase composition of the samples becomes Mg(OH)_2_, and the Mg(OH)_2_ contents of the surface layer and inner core of the MOC samples are 54% and 56%, respectively, while the P 5 amounts are 12% and 15%, respectively. The compressive strength of the samples decreases from 93.2 MPa to 8.1 MPa and reduces by 91.3%, and their flexural strength declines from 16.4 MPa to 1.2 MPa. However, their deterioration process is delayed compared with the samples that were dipped in water continuously for 21 days whose compressive strength is 6.5 MPa. This is primarily ascribed to the fact that during the natural drying process, the water in the immersed samples evaporates, the decomposition of P 5 and the hydration reaction of unreacted active MgO both slow down, and the dried Mg(OH)_2_ may provide the partial mechanical properties, to some extent.

## 1. Introduction

Greenhouse gas emissions are a main contributing factor to global climate change, and CO_2_ accounts for approximately 76% of greenhouse gas emissions. Presently, the cement industry is a major industry that discharges CO_2_ in China [1]. Therefore, the development of new technologies or low-carbon building materials is an effective way to reduce CO_2_ emissions from the cement industry, and it may also help achieve “carbon peak” and “carbon neutralisation” goals [2].

Magnesium oxychloride cement (MOC) is a magnesium building material with a low carbon footprint and excellent performance that is primarily formed by a stirring and curing reaction of light burned magnesia and magnesium chloride solution. Light burned magnesia comes from the calcination of magnesite whose calcination temperature is 750–850 °C, which is significantly lower than that of ordinary cement (1400 °C). Magnesium chloride is a byproduct of preparing potassium chloride from Qinghai Salt Lake. MOC can bond and solidify various solid wastes, and it can also offset CO_2_ from the air through carbon mineralization [3,4,5,6]. Its degradation process is also environmentally friendly. Therefore, it is widely used in fireproof boards, building partitions, door core panels, arts and crafts, transportation packaging boxes, and so on.

Presently, most research on MOC focuses on water resistance, the development of novel composite materials, and its comprehensive utilisation. For example, some modifying agents, such as fly ash, phosphate, organic matter, and hydrophobic agents, have been used to improve the water resistance of MOC by changing the morphology of its hydration products, stabilizing its P 5 (5Mg(OH)_2_·MgCl_2_·8H_2_O), inhibiting the formation of a large number of Mg(OH)_2_ crystals, increasing its compactness, generating amorphous or other types of inorganic gels, and isolating water molecules [7,8,9,10,11]. Studies have added expanded perlite/paraffin into MOC to form a composite with excellent mechanical properties and high thermal insulation and heat storage performance [12]. Studies [13] have also prepared MOC/FSPCM composites which exhibit high heat storage efficiency and good mechanical properties by adding morphologically stabilised phase change material into MOC. Wood chips and/or perlite have been mixed into MOC to produce a composite which can be nailed like natural hardwood and possesses a dry density close to 1.0 [14]. Studies [15] have prepared straw-containing magnesium cement (SMC) composite, and researchers [4] have produced MOC boards that contain wood fiber by adding waste wood from a construction site. Hanna S.B. et al. [16] have mixed silicon carbide (SiC) into MOC, which subsequently formed a grinding stone. Additionally, a low-cost, formaldehyde-free, and high-flame retardant wood adhesive has been fabricated by using MOC inorganic material as raw material [17]. These studies have enriched the basic theory of MOC and expanded the application field of MOC materials.

Durability is important for MOC materials [18,19,20,21]. A study on the durability of MOC is vital to improve the service life and economic benefits of MOC materials. The durability of MOC materials is related to the nature of the material itself and is also closely connected to its service environment. Different service conditions have diverse effects on the properties of MOC materials. Particularly, the outdoor dry–wet cyclic environment, which is a common service condition, has an important impact on materials. Jinjun Xu et al. [22] have studied the physical and mechanical properties of excavated soil-based unfired clay bricks before and after dry–wet cycles. Aline Carvalho et al. [23] have evaluated the environmental durability of press-molded, self-fitting soil-cement blocks with granite waste during standard water immersion, wetting, and drying cycles. However, it is necessary to study the durability of MOC materials in an outdoor dry–wet cycle environment further. Additionally, studies on the deterioration performances and mechanisms of MOC materials are vital to improve the materials’ durability.

The surface layer and inner core of the MOC samples were both investigated to reveal the deterioration process of MOC materials in an outdoor dry–wet cyclic environment while taking the volume effect of MOC into account. The evolution of the macroscopic and microscopic morphology, bulk density, phase composition, and mechanical properties of the surface layer and inner core of the MOC samples during dry–wet cycles was studied, and the deterioration mechanisms of the MOC samples were revealed. This will provide reference for the application of MOC materials in an outdoor service environment.

## 2. Materials and Methods

### 2.1. Principal Raw Materials

The main raw materials used in this study were bischofite from Golmud (Qinghai Province, China) and light-burned magnesia from Haicheng (Liaoning Province, China). Their chemical compositions are listed in Table 1 and Table 2.

### 2.2. Preparation of MOC Samples

**Figure 1:**
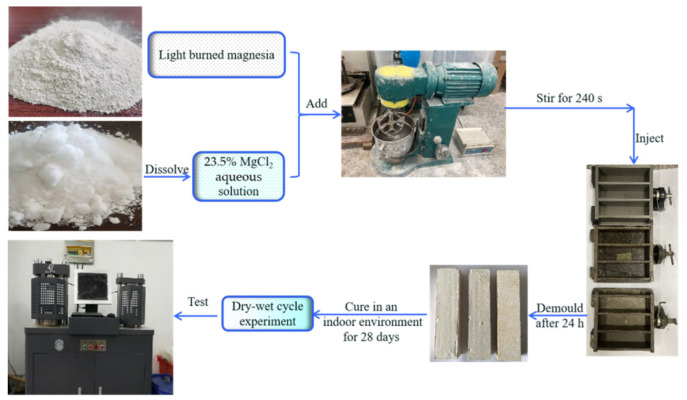
Flowchart showing the fabrication process of the MOC samples.

Premade magnesium chloride aqueous solution with a mass fraction of 23.5% was available. The MOC samples were prepared according to the molar ratio of active MgO, MgCl_2_, and H_2_O at 7.2:1:16. Light-burned magnesium oxide powder and the premade 23.5 wt.% MgCl_2_ aqueous solution were mixed and stirred evenly for approximately 240 s until they formed a slurry body, which was then injected into a 40 mm × 40 mm × 160 mm mould to harden. The samples were removed from the mold after 24 h and then cured naturally in an indoor environment (temperature = 23 ± 1 °C, relative humidity = 40 ± 2%) for 28 days. The corresponding sample was marked “a”. The preparation process of the samples is shown in Figure 1. 

### 2.3. Dry–Wet Cyclic Experiments

**Figure 2:**
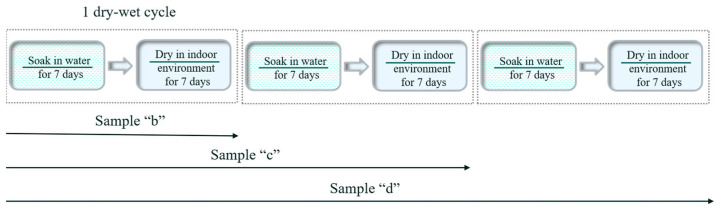
Schematic diagram of dry–wet cyclic experiments for the MOC samples and their corresponding sample labels.

The MOC samples that were cured in an indoor environment for 28 days were soaked in distilled water for 7 days and then taken out and dried naturally in an indoor environment (temperature 23 ± 2 °C, humidity 40 ± 2%) for 7 days. This was a dry–wet cycle period (14 days in total). Sample “b” experienced 1 dry–wet cycle, and sample “c” experienced 2 dry–wet cycles, which lasted 28 days. When the samples were subjected to 3 dry–wet cycles, they were named as “d”, totaling 42 days. By that time, the samples had already been seriously warped and deformed, and the dry–wet cyclic test was stopped. The dry–wet cyclic experiment as well as the corresponding sample labels are presented in Figure 2.

### 2.4. Characterization

A CP513 electronic balance was used to test the quality of the MOC samples after different dry–wet cycles. As for each scheme, i.e., samples “a”, “b”, “c”, and “d”, 6 parallel specimens were taken, and each specimen was weighed 5 times. The obtained average value M (unit: g) was the mass of the samples. Depending on Equation (1), the bulk density (g/cm^3^) was calculated, in which 256 was the sample volume (cm^3^).

(1) $$\mathrm{Bulk}\;\mathrm{density}=\frac{M}{256}$$

The microscopic morphology of the MOC samples was studied by using a field emission scanning electron microscope (FE-SEM, SU8010, Hitachi High-Tech, Tokyo, Japan) after they were coated with gold. An X-ray diffractometer (XRD, D8 Discover, Bruker, Karlsruhe, Germany) with a Cu Kα radiation source was applied to identify the phase composition of the MOC samples, which was performed with a step length of 0.02° and a dwell time of 2 s in a scan range from 20° to 80° (in 2 θ) at 40 kV and 40 mA. The thermal stability of the MOC samples after different dry–wet cycles were tested using a simultaneous thermal analyser (TG-DSC, STA449F3, NETZSCH, Selb, Bavaria, Germany) from room temperature to 800 °C at a heating rate of 10 °C/min under the protection of the nitrogen atmosphere. A Fourier transform infrared spectrometer (FT-IR, Nexus, Thermo-Nicolet, Madison, New York, NY, USA) was used to analyze the chemical functional groups of the MOC samples in a range from 400–4000 cm^−1^.

The compressive and flexural strengths of the MOC samples were tested at a loading rate of 2400 N/s according to GB/T17671-2020 of the “Cement Rubber Sand Strength Test Method” (ISO method) using a microelectromechanical electrohydraulic servo-pressure testing machine (model HYE-300B-D, Beijing Sanyuweiye Testing Machine Co., Ltd, Beijing, China). To acquire the reliable compressive and flexural strengths, each scheme (samples “a”, “b”, “c”, and “d”) was repeated 6 times, and the average values after removing any abnormal values from the original measured values were the compressive and flexural strengths of the samples. Their standard deviations were also obtained.

**Figure 3:**
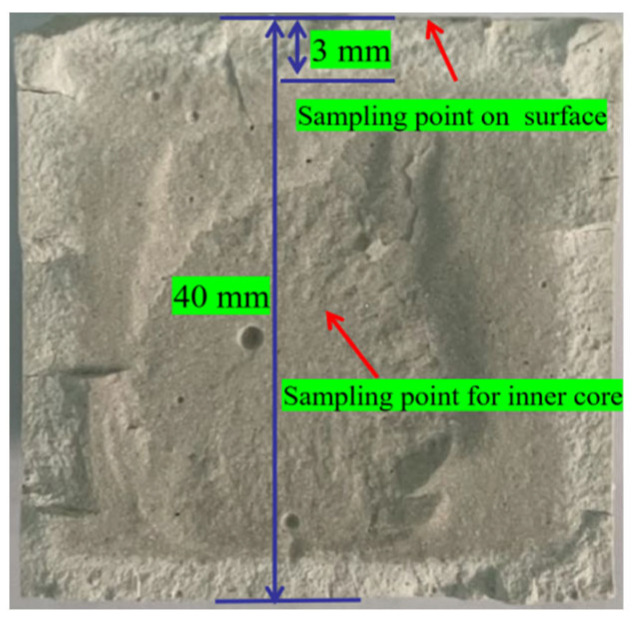
Measurement of the water erosion depth and sampling points.

Additionally, a Vernier calliper was used to measure the water erosion depth in the cross-section of the sample, and the sampling point was marked (Figure 3). For example, the water erosion depth of sample “b” was found to be 3 mm.

## 3. Results

### 3.1. Macroscopic Morphology

**Figure 4:**
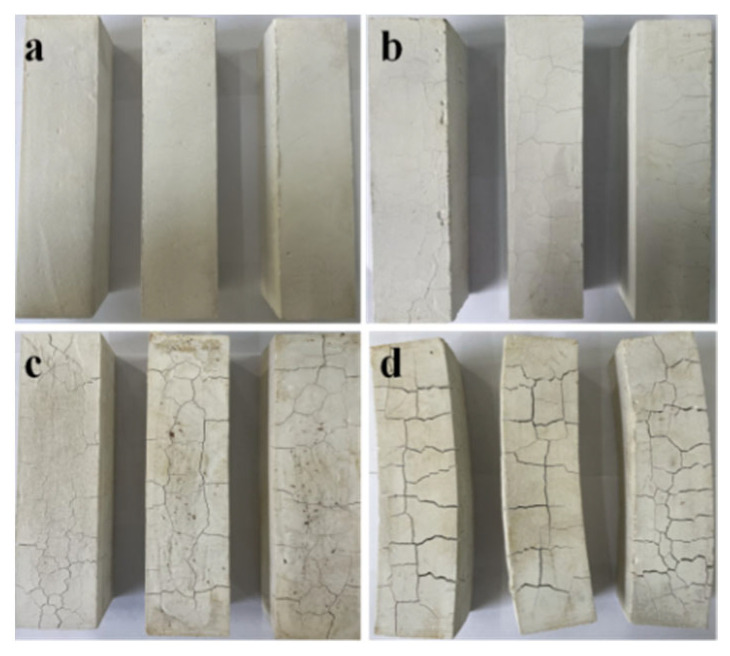
Macro morphology of the MOC samples after different dry–wet cycles: (**a**) without dry–wet cycles; (**b**) one time; (**c**) two times; (**d**) three times.

Figure 4 shows the macro morphology of samples “a”, “b”, “c”, and “d”. It can be seen from Figure 4 that the surface of sample “a” cured in an indoor environment for 28 days is milky white, without cracks, and relatively compact. However, after different dry–wet cycles, the samples exhibit diverse macroscopic morphologies. Among them, for sample “b”, which experienced one dry–wet cycle, some small cracks appear on its surface, which are mainly attributed to volume expansion caused by phase transformation. The cracks on the surface of sample “c” become much wider. The cracks of sample “d” further increase and widen, and the sample warps and deforms. At the same time, the edges and corners of sample “d” fall off, indicating that the sample almost loses efficacy.

### 3.2. Depth of Water Erosion and Bulk Density

**Figure 5:**
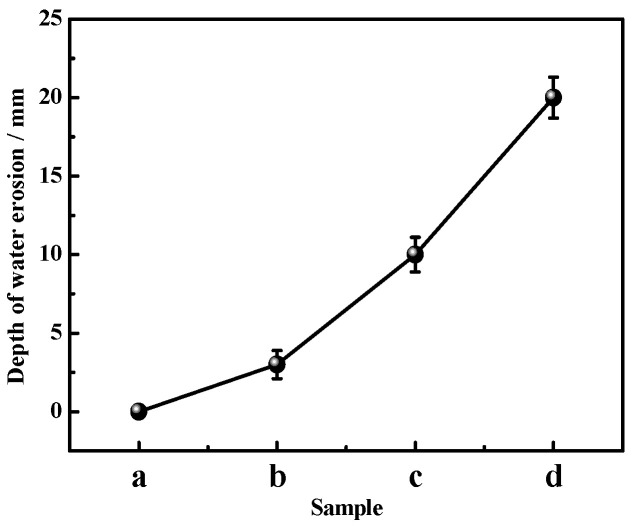
Water erosion depth of the MOC samples after different dry–wet cycles: (a) without dry–wet cycles; (b) one time; (c) two times; (d) three times.

Figure 5 presents the depth of water entering the samples after different dry–wet cycles. It can be observed from Figure 5 that as the cycle number increases, the depth of water getting into the sample gradually augments. After three dry–wet cycles, the water completely reaches the interior of the sample, and according to Figure 4, the sample basically loses its integrity.

**Figure 6:**
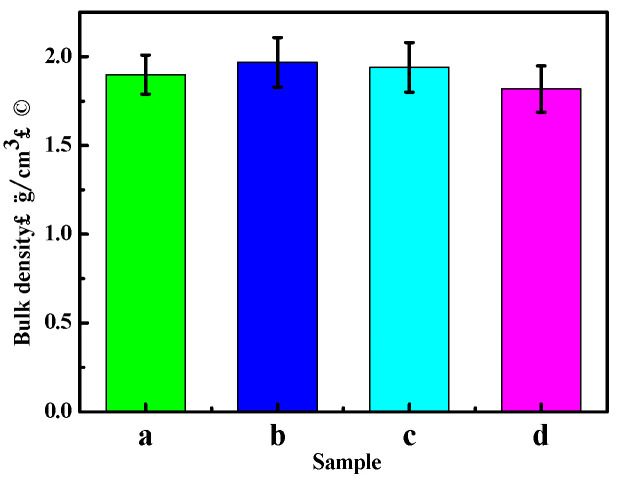
Bulk density of the MOC samples after different dry–wet cycles: (a) without dry–wet cycles; (b) one time; (c) two times; (d) three times.

Figure 6 displays the variation in the bulk density of the sample with the dry–wet cycle numbers. As shown in Figure 6, the bulk density of the sample first aggrandizes and then decreases with increasing cycle times. This may be because when the cycle number augments, the water molecules in the sample gradually increase, resulting in a slight boost in the bulk density. However, when the number of dry–wet cycle increases, the bulk density declines due to the sample’s change in structure. Bulk density reflects the compactness of the sample’s interior, to some extent. Therefore, a reduction in bulk density indicates that the inside of the sample becomes loose, which may reduce the mechanical properties of the sample.

### 3.3. Mechanical Properties

**Figure 7:**
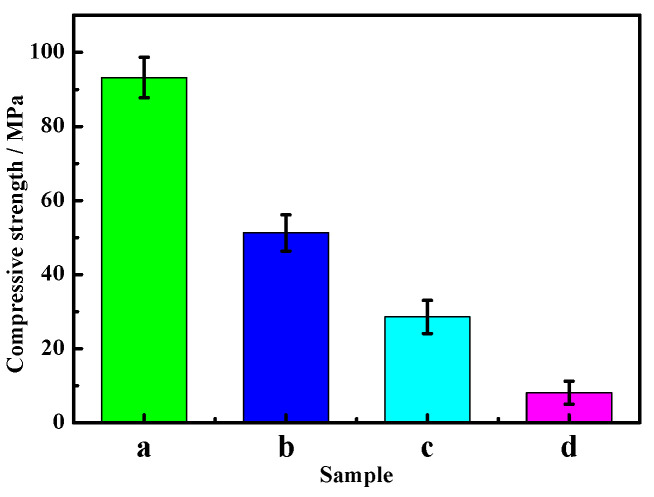
Compressive strength of the MOC samples after different dry–wet cycles: (a) without dry–wet cycles; (b) one time; (c) two times; (d) three times.

**Figure 8:**
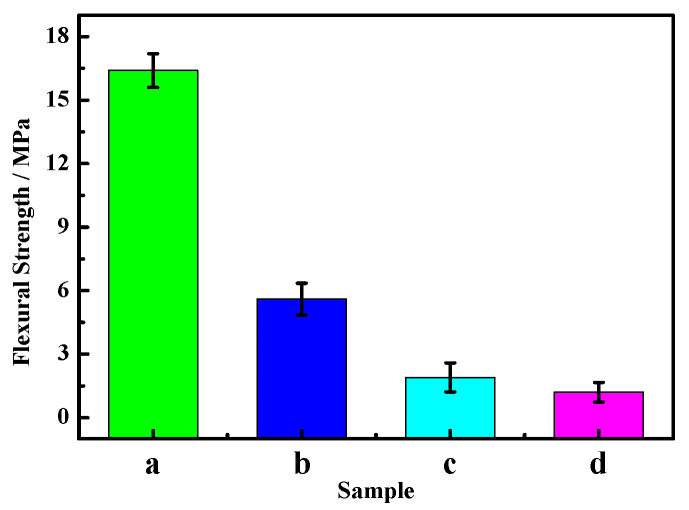
Flexural strength of the MOC samples after different dry–wet cycles: (a) without dry–wet cycles; (b) one time; (c) two times; (d) three times.

Figure 7 and Figure 8 show the variation in the compressive and flexural strengths of the MOC samples according to the number of dry–wet cycles. As can be seen from Figure 7 and Figure 8, both the compressive strength and flexural strength decrease significantly as the cycle number increases, and the reduced degree is larger. After three dry–wet cycles, the compressive strength of MOC is only 8.1 MPa, and the flexural strength decreases to 1.2 MPa. This indicates that dry–wet cycle times have influence the mechanical properties of MOC, and its mechanical properties basically disappear after three dry–wet cycles.

### 3.4. Microscopic Morphology

**Figure 9:**
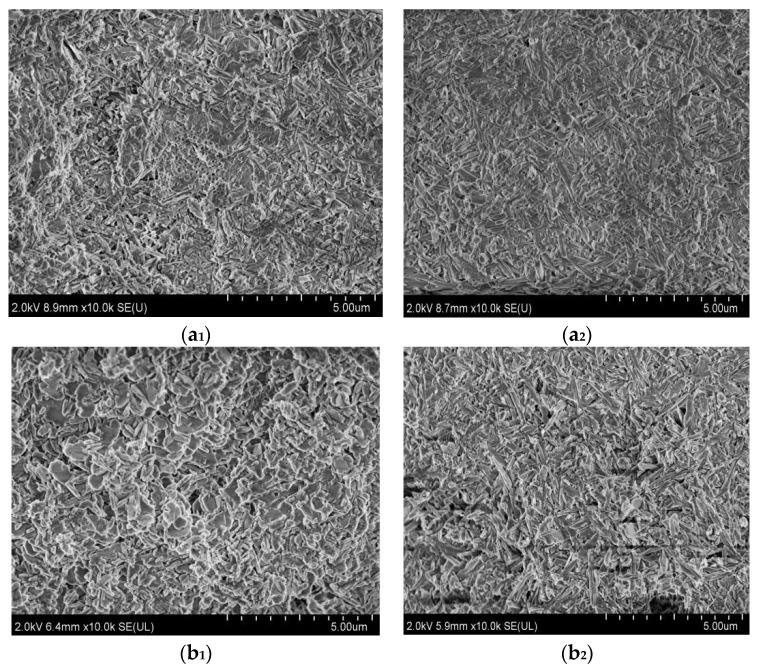

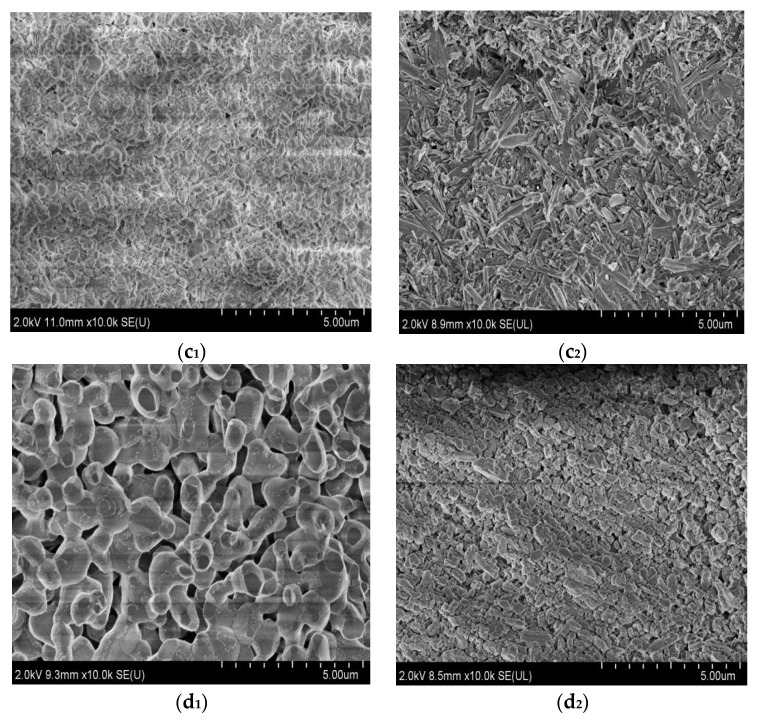
Microscopic morphologies of the surfaces (**a_1_**–**d_1_**) and inner cores (**a_2_**–**d_2_**) of the MOC samples after different dry–wet cycles: (**a_1_**,**a_2_**) without dry–wet cycles; (**b_1_**,**b_2_**) one time; (**c_1_**,**c_2_**) two times; (**d_1_**,**d_2_**) three times. (**a_1_**) Surface layer of sample “a”; (**a_2_**) inner core of sample “a”; (**b_1_**) surface layer of sample “b”; (**b_2_**) inner core of sample “b”; (**c_1_**) surface layer of sample “c”; (**c_2_**) inner core of sample “c”; (**d_1_**) surface layer of sample “d”; (**d_2_**) inner core of sample “d”.

Figure 9 displays the micro morphology of the surfaces and inner cores of samples “a”, “b”, “c”, and “d”. As can be observed from Figure 9, there is no obvious difference in the morphology for the surface and inner core of sample “a”. They are both gelatinous, short, and rod-like, and they exist crisscross and make the sample relatively dense. The inner core of sample “b” is still gelatinous, short, and rod-like, but some micro−flake morphology appears on its surface, which is relatively loose, indicating that the surface microscopic morphology of sample “b” changes after one dry–wet cycle. Sample “c” possesses more micro-flake morphology on its surface, and some micro-flake morphology also occurs in its inner core. Both the surface and inner core of sample “d” are occupied by micro-flake morphology; hence, the microstructures of sample “d” are very loose and porous, suggesting that after three dry–wet cycles, water molecules get inside the sample, the gel state and short, rod-like morphology of the MOC sample almost disappears, and the sample loses its original structure.

### 3.5. Phase Composition

**Figure 10:**
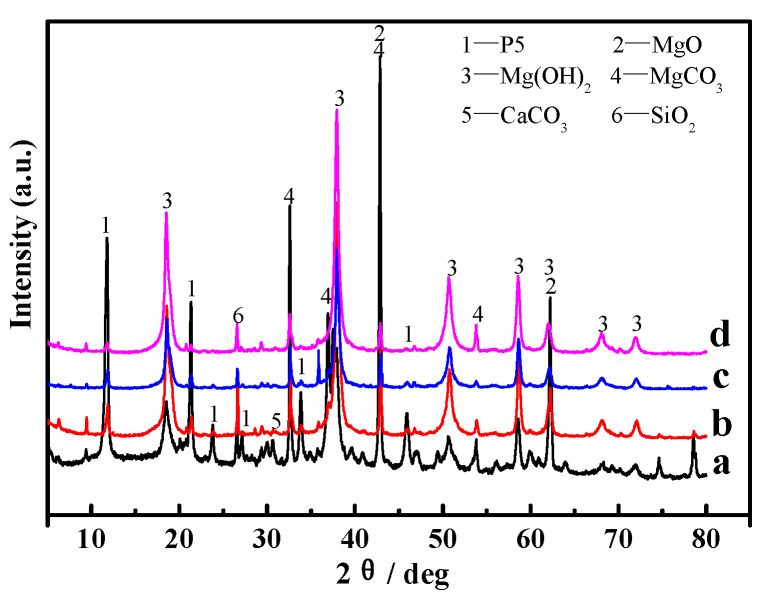
XRD patterns of the surface layers of the MOC samples after different dry–wet cycles: (a) without dry–wet cycles; (b) one time; (c) two times; (d) three times.

**Figure 11:**
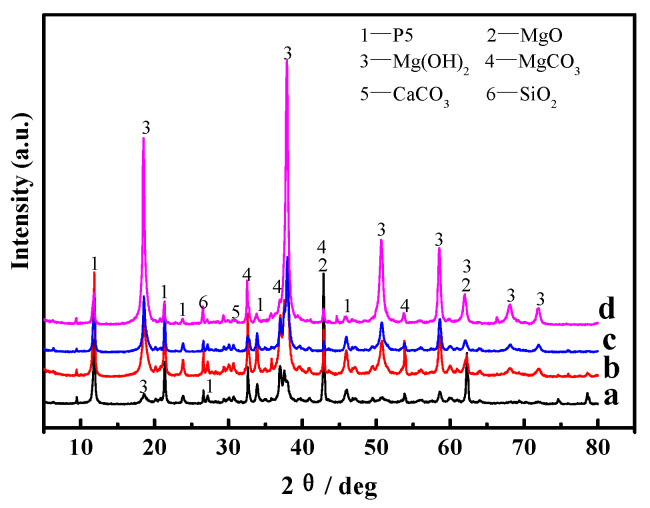
XRD patterns of the inner cores of the MOC samples after different dry–wet cycles: (a) without dry–wet cycles; (b) one time; (c) two times; (d) three times.

Figure 10 and Figure 11 present the XRD patterns of the surface layers and inner cores of samples “a”, “b”, “c”, and “d”. The main phases of the samples were analyzed semi-quantitatively using tHighScorePlus4.9 software, and the corresponding analytical results are listed in Table 3. As can be seen from Figure 10 and Figure 11, diffraction peaks of MgO, SiO_2_, MgCO_3_, and CaCO_3_ appear on the surface layers and the inner cores of the samples. All of them derive from the light-burned magnesia oxide in the raw material, where MgO does not participate in the reaction in the light-burned magnesia powder.

It can be found from Figure 10 and Table 3 that the diffraction peaks of 5Mg(OH)_2_·MgCl_2_·8H_2_O (abbreviated as phase 5·1·8, P 5) in sample “a” are higher and sharper, indicating more P 5 content. The diffraction peaks of MgO are also obvious; meanwhile, there are some weak diffraction peaks belonging to Mg(OH)_2_, suggesting that sample “a” contains unreacted MgO, and there is a partial MgO transformation to Mg(OH)_2_. In samples “b” and “c”, the diffraction peaks of P 5 and MgO gradually decrease, and they almost disappear in sample “d”. This demonstrates that the main phase of the MOC sample changes to Mg(OH)_2_ after three dry–wet cycles.

The XRD patterns of the inner cores of samples “a”, “b”, “c”, and “d” are displayed in Figure 11. It can be seen from Figure 11 that the P 5 in samples “a” and “b” barely change, but the MgO diffraction peaks in sample “b” slightly decrease, while the Mg(OH)_2_ diffraction peaks increase. With increasing dry–wet cycle numbers, the content of P 5 and the MgO phase in the sample gradually reduce, while the content of the Mg(OH)_2_ phase further augments, as shown in Figure 11c,d. Among them, the MgO content in sample “d” reduces to zero, and the content of the Mg(OH)_2_ phase is close to that in the outer layer (see Table 3). Both the inner and outer layers of sample “d” are mainly composed of Mg(OH)_2_, which indicates that water molecules completely enter into the inner core of the sample, giving rise to the change in the main phase composition. While P 5 is the major strength phase, a decrease in its content will inevitably lead to a decline in the mechanical properties of MOC materials, as shown in Figure 7 and Figure 8.

### 3.6. TG-DSC Analyses

**Figure 12:**
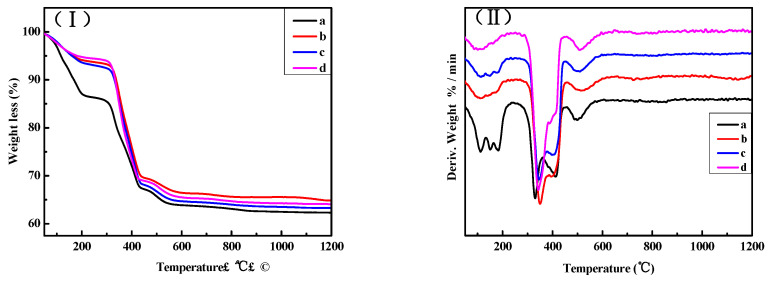
TG curves (**I**) and DTG curves (**II**) of the surface layers of the MOC samples after different dry–wet cycles: (a) without dry–wet cycles; (b) one time; (c) two times; (d) three times.

Figure 12 shows the TG and DTG curves of the surface layers of samples “a”, “b”, “c”, and “d”. As can be observed from Figure 12, the total weight loss in sample “a” is 22.81% in the range from 0 to 350 °C, and in the corresponding DTG curves, there are three endothermic peaks that appear near 111 °C, 150 °C, and 180 °C. This indicates that 22.81% of the weight loss is caused by the gradual loss of eight water molecules from 5Mg(OH)_2_·MgCl_2_·8H_2_O. In the DTG curve of sample “a”, endothermic peaks also appear near 330 °C, 400 °C, and 500 °C, among which the endothermic peak at 330 °C is the strongest, representing the enhanced decomposition peak of Mg(OH)_2_ and P 5 [24,25]. The weight loss in samples “b”, “c”, and “d” is similar and in a range between 0–350 °C, which is about 13% and attributed to the decomposition of P 5. In the corresponding DTG curve, the endothermic peaks near 111 °C, 150 °C, and 180 °C markedly weaken, indicating that the content of P 5 significantly reduces. The peak at 330 °C enhances in an obvious way, suggesting that there is increasing Mg(OH)_2_ content. This is consistent with the results of XRD for the surface layers.

**Figure 13:**
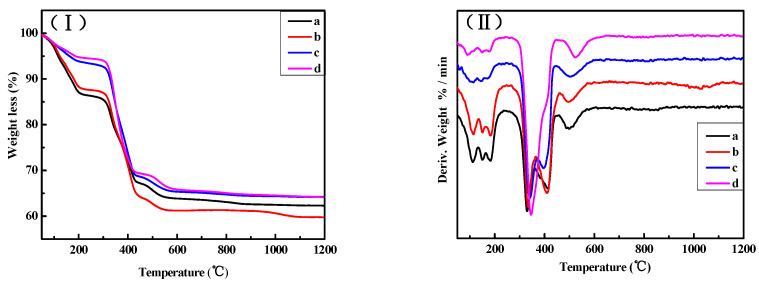
TG curves (**I**) and DTG curves (**II**) of the inner cores of the MOC samples after different dry–wet cycles: (a) without dry–wet cycles; (b) one time; (c) two times; (d) three times.

The TG and DTG curves of the inner cores of samples “a”, “b”, “c”, and “d” are presented in Figure 13. As can be seen from Figure 13, in the range between 0–350 °C, the weight loss of sample “a” is approximately 22%, while that of sample “b” is about 15%. At the same time, endothermic peaks appear around 111 °C, 150 °C, and 180 °C; however, the endothermic peaks in sample “b” are significantly weaker, indicating that compared with the inner core of sample “a”, the P 5 content in sample “b” reduces. In the range from 0–320 °C, the weight loss of samples “c” and “d” is approximately 6%. The endothermic peaks near 111 °C, 150 °C, and 180 °C markedly weaken, but the endothermic peak at 330 °C notably enhances, especially for sample “d”. This indicates that with an augmented cycle number, the content of P 5 in samples “c” and “d” decreases, yet the content of Mg(OH)_2_ increases.

### 3.7. FT-IR Spectra Analyses

**Figure 14:**
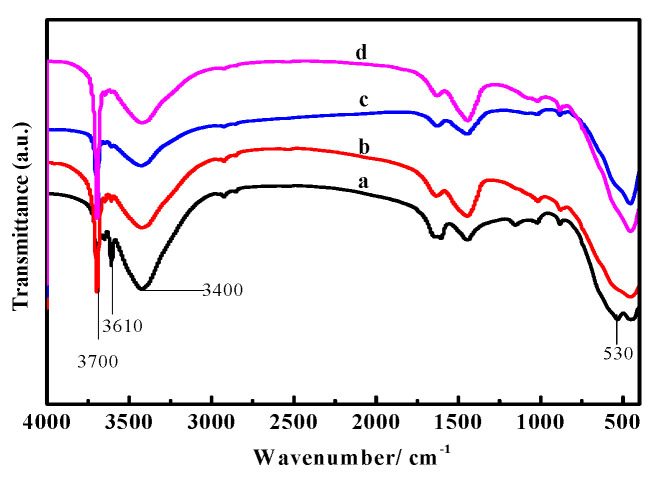
FT-IR spectra of the surface layer of the MOC samples after different dry–wet cycles: (a) without dry–wet cycles; (b) one time; (c) two times; (d) three times.

**Figure 15:**
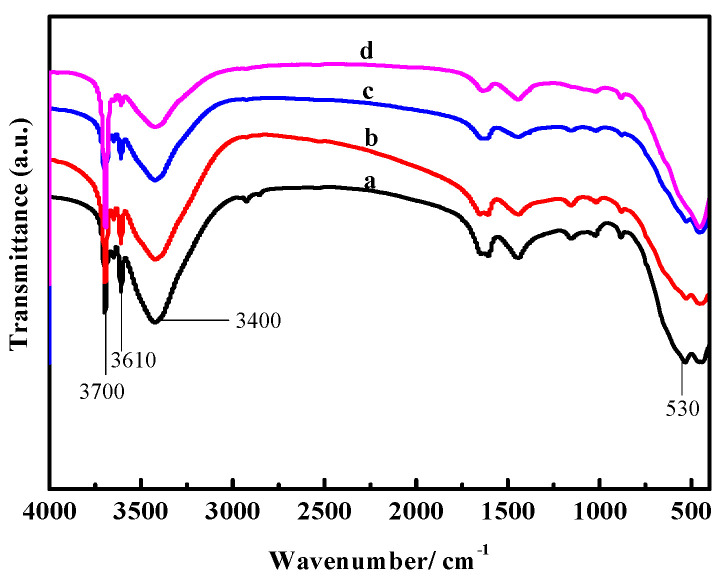
FT-IR spectra of the inner cores of the MOC samples after different dry–wet cycles: (a) without dry–wet cycles; (b) one time; (c) two times; (d) three times.

Figure 14 and Figure 15 show the FT-IR spectra of the surface layers and inner cores of samples “a”, “b”, “c”, and “d”. There is a strong absorption band in a range from 700 to 400 cm^−1^, which is the stretching vibration absorption band of the Mg-O bond and the characteristic peak of MgO. The absorption peak near 1500 cm^−1^ is the characteristic peak of carbonate. The characteristic peak of coordination water molecule vibration is between 3330 cm^−1^ and 3500 cm^−1^. The sharp absorption peaks at 3670 cm^−1^ and 3610 cm^−1^ are the nonaqueous hydroxyl (−OH) stretching vibration peak superimposed on crystalline water, which belongs to the characteristic peak of P 5. The peak at 3700 cm^−1^ is the characteristic peak of Mg(OH)_2_ [26,27].

It can be seen from Figure 14 that in sample “a”, the characteristic peaks at 530 cm^−1^, 3670 cm^−1^, and 3610 cm^−1^ are stronger, while the peaks at 3700 cm^−1^ are weaker, indicating that the content of P 5 and MgO in sample “a” is higher, yet the content of Mg(OH)_2_ is lower. With an increased number of dry–wet cycles, the characteristic peaks at 530 cm^−1^, 3670 cm^−1^, and 3610 cm^−1^ gradually weaken in samples “b”, “c”, and “d”, respectively, while the peaks at 3700 cm^−1^ strengthen, suggesting that the MgO and P 5 content slowly decrease with the augmented cycle number, but the content of Mg(OH)_2_ increases. This is in accordance with the results from the XRD and TG-DSC analyses.

Figure 15 shows that the peaks near 3670 cm^−1^ and 3610 cm^−1^ in samples “a” and “b”, respectively, are stronger than those in samples “c” and “d”, indicating less P 5 content in samples “c” and “d”. The peaks at 3700 cm^−1^ in samples “c” and “d” are stronger compared with samples “a” and “b”, especially in sample “d”, indicating that the content of Mg(OH)_2_ in sample “d” is larger. As Figure 14 and Figure 15 show, after three dry–wet cycles, water molecules reach the inner core of the sample, causing the transformation of the overall phase of the sample.

## 4. Discussion

Magnesium oxychloride cement (MOC) is an inorganic, nonmetallic material that possesses a low carbon footprint, low alkalinity, and strength [28,29]. Its preparation process includes adding light-burned magnesia powder to MgCl_2_ aqueous solution, which is stirred into a paste and then injected into a mold to form. During this process, the active MgO first dissolve into Mg^2+^ and OH^−^; subsequently, Mg^2+^, Cl^−^, and H_2_O in the slurry directly react to form 5Mg(OH)_2_·MgCl_2_·8H_2_O (abbreviated as 5·1·8 phase, P 5) (see Equation (3)), 3Mg(OH)_2_·MgCl_2_·8H_2_O (abbreviated as 3·1·8 phase, P 3) (Equation (2)), and gel Mg(OH)_2_ (Equation (4)). When the molar ratio of active MgO/MgCl_2_ is greater than six, the obtained main products are P 5 and gel Mg(OH)_2_ [30,31,32,33]. In this study, the MOC samples were prepared according to the molar ratio of active MgO, MgCl_2_ and H_2_O, which is 7.2:1:16; therefore, the molar ratio of active MgO/MgCl_2_ is higher than six. Thus, the major phases of the MOC samples are P 5 and gel Mg(OH)_2_, as shown in Figure 10 and Figure 11. Additionally, their primary microscopic morphology is rod-like and in a gel state with a relatively dense microstructure, as seen in Figure 9(a_1_,a_2_). Therefore, sample “a” without a dry–wet cycle exhibits better compressive and flexural strengths due to the presence of the main strength phase (P 5) and its dense structure.

3MgO + MgCl_2_ + 11H_2_O → 3Mg(OH)_2_·MgCl_2_·8H_2_O (2)

5MgO + MgCl_2_ + 13 H_2_O → 5Mg(OH)_2_·MgCl_2_·8H_2_O (3)

MgO + H_2_O → Mg(OH)_2_ (Gel) (4)

The dry–wet cycle numbers significantly influence the deterioration of the MOC samples. After one dry–wet cycle, i.e., for sample “b”, the depth of water entering into the sample is 3 mm. During the 7-day immersion in water, water molecules enter the surface layer of the sample, leading to the decomposition of the main strength phase P 5 of the sample’s surface layer and the generation of Mg(OH)_2_ and MgCl_2_. Among them, MgCl_2_ dissolves in water (Equation (5)) and disappears. At the same time, the unreacted active MgO undergoes a hydration reaction, forming Mg(OH)_2_, as shown in Equation (6). Microcracks appear on the surface of sample “b” because of the larger volume of Mg(OH)_2_ compared with that of MgO, as illustrated in Figure 4b. As a result, the mechanical properties of sample “b” decrease (Figure 7b and Figure 8b). The “soundness”, which is a term used to describe cement paste specimens that do not exhibit cracks, disintegration, or other flaws that result from excessive volume changes, exhibits a significant influence on the properties of the MOC samples, so it is necessary to measure the “soundness” in future research [34,35].

5Mg(OH)_2_·MgCl_2_·8H_2_O + H_2_O → 5Mg(OH)_2_ + MgCl_2_ (Loss) (5)

active MgO + H_2_O → Mg(OH)_2_ (6)

**Figure 16:**
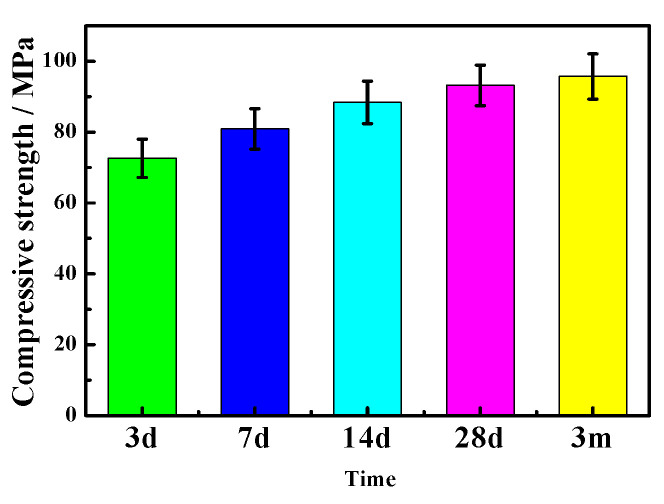
Compressive strength of the MOC samples cured naturally for different time in an indoor environment.

**Figure 17:**
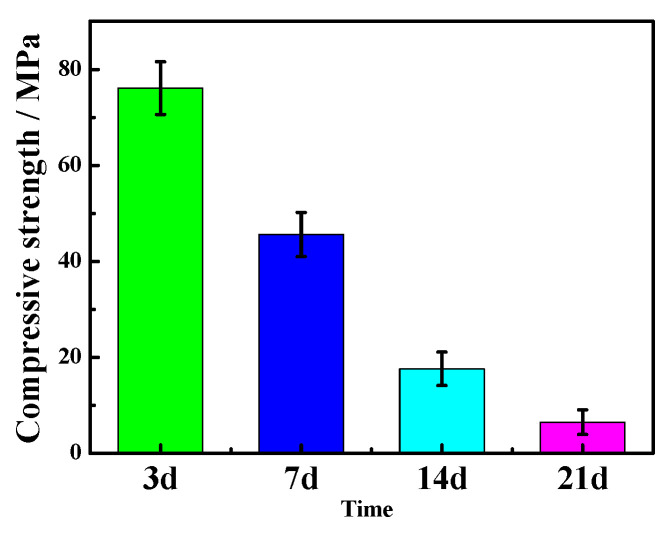
Compressive strength of the MOC specimens immersed in water for different times.

During indoor natural drying for 7 days, the water molecules’ infiltration into the specimen stops, i.e., its damage to the inside of the sample ceases. In addition, reaction (5) and reaction (6) are slowed down due to the evaporation of the water molecules, which lowers the destruction of the soaked part of the samples. These are helpful to postpone the overall failure of the sample. As a consequence, the compressive strength of sample “b” (51.25 MPa) is much lower than those cured naturally for 28 days and 3 months in an indoor environment, which are 93.2 MPa and 95.7 MPa, respectively, as shown in Figure 16, but it is still slightly higher than that soaked in water for 7 days (45.6 MPa), as presented in Figure 17. This indicates that the natural drying process during the dry–wet cycle is conducive to inhibiting a decrease in the mechanical properties of the MOC samples, to some extent, which may be attributed to a slower deterioration process and the partial mechanical properties provided by the drying Mg(OH)_2_. This is different from the literature [36], in which the crystal crystallization and crystal transformation caused by sodium sulfate from the corrosive solution can also give rise to the deterioration of concrete during the drying process.

After two dry–wet cycles (sample “c”), the water erosion depth further increases to 10 mm. When sample “c” is immersed in water for 7 days once more, reaction (5) and reaction (6) continue to take place, giving rise to the decomposition of a large amount of P 5. This results in the gel state and short, rod-like morphology becoming a micro-flake morphology on the surface. Meanwhile, some micro-flake morphology also appears in the inner core owing to the increase in the water immersion depth. Furthermore, because of the boost in the content of Mg(OH)_2_, the volume expansion is more serious and augments cracks on the surface of sample “c”. As a result, the mechanical properties of sample “c” further decline due to the reduction in the main strength phase P 5, the loosening of the micro-flake structures, as well as the increasing and widening of the cracks. However, when it is dried again for 7 days, the water erosion process intermits, and the reaction of Equations (5) and (6) slows down once more; as a result, the compressive strength of sample “c” (28.58 MPa) is still higher than that of the sample soaked in water for 14 days, as shown in Figure 17. The compressive strength of the sample dipped in water for 14 days is 17.6 MPa.

Sample “d” was studied after three dry–wet cycles. At this time, water molecules arrive at the inner core of the sample. As a result, the sample suffers from serious warping and deformation; the original gelatinous and short, rod-like morphology disappears due to the decomposition of a number of main strength phase P 5 and hydration reactions of unreacted active MgO; and a micro-flake morphology is formed from the outside to the inside of the sample. Meanwhile, the whole sample is mainly composed of Mg(OH)_2_. Therefore, the mechanical properties of the sample “d” almost disappear.

In conclusion, in an outdoor dry–wet cyclic environment, samples slowly deform, cracks generate and widen inch by inch, and the morphology and phase composition change due to the gradual invasion of water molecules into the sample, causing a decline in mechanical properties and increasingly deteriorated MOC materials. However, the natural drying process is helpful to slow down the decrease in the mechanical properties of MOC, to some extent, which delays the failure of MOC materials so that the service life of MOC materials in an outdoor dry and wet cycle environment is longer than that in full immersion in water.

## 5. Conclusions

The service environment is a key factor to consider when studying the durability of MOC materials because it has a significant impact on the structure and performance of the MOC samples. The outdoor dry–wet cycle environment is a relatively common service environment; therefore, it is of great practical significance to study the deterioration process of MOC materials in a dry and wet cycle environment.

(1) The dry–wet cyclic numbers exhibit an obvious influence on the macro- and micro-structures and properties of the MOC samples. As the dry–wet cycle numbers increase, water molecules gradually intrude into the inner core of the sample, resulting in the hydrolysis of P 5 and the hydration reaction of unreacted active MgO. This leads to cracks, warping, and deformation of the sample. After three dry–wet cycles, the micro-morphology changes from a gel state and short and rod-like to micro-flake shapes which are relatively loose and porous. The phase composition is transformed from P 5 to Mg(OH)_2_, in which the content of P 5 is only 12% and 15% for the surface layers and inner cores of the MOC samples, respectively, while the amount of Mg(OH)_2_ is 54% and 56%, respectively. These give rise to a decline in the compressive strength, from 93.2 MPa to 8.1 MPa, and a decrease of 92.7% in the flexural strength, which gradually deteriorates the MOC materials.
(2) The natural drying process in the dry–wet cycle slows down the deterioration reaction of the sample, and the dried Mg(OH)_2_ may provide part of the mechanical properties for sample, which delay the deterioration process of MOC in a dry–wet cyclic service environment compared with the samples dipped continuously in the water for 21 days whose compressive strength is 6.5 MPa.
(3) Further research is still needed to improve the durability of MOC materials in the dry–wet cyclic environment by internal modification, such as doping fly ash, phosphoric acid, FeSO_4_ and nano SiO_2_, and external surface treatment, such as spraying hydrophobic organic coatings.

## Figures and Tables

**Table 1:** Chemical composition of bischofite.

Composition	MgCl_2_	NaCl	MgSO_4_	KCl	CaCl_2_	Water-Insoluble Matter
Content (wt.%)	44.90	0.13	0.06	0.01	0.03	0.27

**Table 2:** Chemical composition of light-burned magnesia.

Composition	MgO	MgCO_3_	CaCO_3_	f-CaO	Acid-Insoluble Matter
Content (wt.%)	69.52	19.80	1.34	0.38	8.41

**Table 3:** Content of the main phase for the surface layers and inner cores of the MOC samples after different dry–wet cycles: (a) without dry–wet cycles; (b) one time; (c) two times; (d) three times.

Sampling Points	Samples	5·1·8 Phase/%	MgO/%	Mg(OH)_2_/%
Surface layer	a	43	25	8
	b	26	14	39
	c	22	8	45
	d	12	0	54
Inner core	a	45	24	9
	b	44	19	21
	c	32	9	40
	d	15	0	56

## Data Availability

The data presented in this study are available upon request from the corresponding author and the first author.
